# Controls and Adaptive Management of Nitrification in Agricultural Soils

**DOI:** 10.3389/fmicb.2019.01931

**Published:** 2019-08-30

**Authors:** Jeanette Norton, Yang Ouyang

**Affiliations:** ^1^Department of Plants, Soils and Climate, Utah State University, Logan, UT, United States; ^2^Department of Microbiology and Plant Biology, Institute of Environmental Genomics, University of Oklahoma, Norman, OK, United States

**Keywords:** nitrification, global change, ammonia oxidizers, nitrite oxidizers, biological nitrification inhibition, agricultural management

## Abstract

Agriculture is responsible for over half of the input of reactive nitrogen (N) to terrestrial systems; however improving N availability remains the primary management technique to increase crop yields in most regions. In the majority of agricultural soils, ammonium is rapidly converted to nitrate by nitrification, which increases the mobility of N through the soil matrix, strongly influencing N retention in the system. Decreasing nitrification through management is desirable to decrease N losses and increase N fertilizer use efficiency. We review the controlling factors on the rate and extent of nitrification in agricultural soils from temperate regions including substrate supply, environmental conditions, abundance and diversity of nitrifiers and plant and microbial interactions with nitrifiers. Approaches to the management of nitrification include those that control ammonium substrate availability and those that inhibit nitrifiers directly. Strategies for controlling ammonium substrate availability include timing of fertilization to coincide with rapid plant update, formulation of fertilizers for slow release or with inhibitors, keeping plant growing continuously to assimilate N, and intensify internal N cycling (immobilization). Another effective strategy is to inhibit nitrifiers directly with either synthetic or biological nitrification inhibitors. Commercial nitrification inhibitors are effective but their use is complicated by a changing climate and by organic management requirements. The interactions of the nitrifying organisms with plants or microbes producing biological nitrification inhibitors is a promising approach but just beginning to be critically examined. Climate smart agriculture will need to carefully consider optimized seasonal timing for these strategies to remain effective management tools.

## Introduction

Human activities have dramatically altered the global nitrogen (N) cycle by increasing the amount of reactive N in the biosphere (Kaiser, 2001; Fowler et al., 2013). The anthropogenic inputs of industrially produced N fertilizers and N fixation by crops now exceed the natural N inputs to terrestrial systems (Galloway and Cowling, 2002; Schlesinger, 2009; Fowler et al., 2013). Yet the N use efficiency (NUE) of our fertilizers in agricultural systems remains quite low, typically only about 50% or less of fertilizer N applied is taken up by the crop during the growing season (Raun and Schepers, 2008; Cavigelli et al., 2012). A better understanding of N cycling in agroecosystems is essential for intensifying sustainable food production while decreasing negative environmental impacts. Overall, improved management of nitrification may increase the NUE of fertilization while reducing the transport of reactive N to rivers and groundwater and the emissions of greenhouse gases especially nitrous oxide (N_2_O) (Smith et al., 2008; Robertson and Vitousek, 2009). These are important considerations for agricultural and environmental policy especially as global climate change intensifies (Schlesinger, 2009; Cavigelli et al., 2012; Robertson et al., 2014).

**Figure 1:**
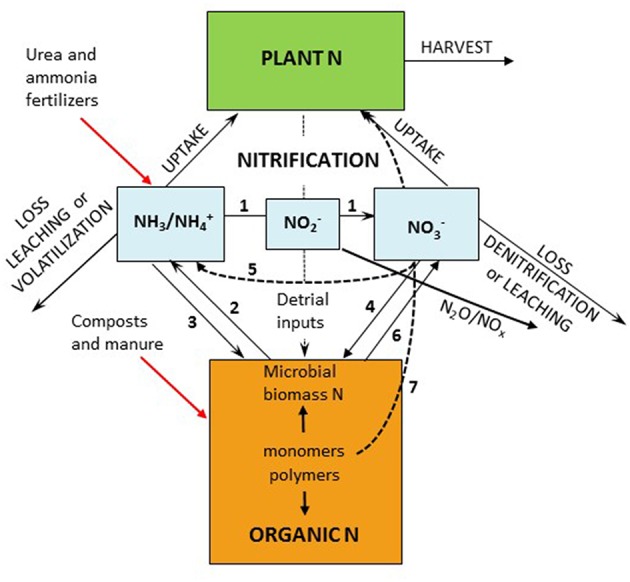
The soil internal nitrogen cycle with (1) nitrification (including comammox), (2) mineralization (ammonification), (3) ammonium immobilization, (4) nitrate immobilization, (5) dissimilatory ${\text{NO}}_{3}^{-}$ reduction to NH_3_ (DNRA), (6) hetrotrophic nitrification, and (7) plant uptake of monomers.

Agriculture is responsible for over half of the input of reactive N to terrestrial systems; however improving N availability through fertilization remains a primary management technique to increase crop yields in most regions. N fertility management is inherently complex because available N is temporally and spatially dynamic and subject to high rates of loss through diverse pathways. Mobility and availability of N from fertilizers and organic sources is the result of microbial enzymatic processes especially mineralization and nitrification operating within the physical and chemical constraints of the soil matrix (Figure 1). In many agricultural systems, large amounts of fertilizer N are lost from the root zone as nitrate through leaching and denitrification (Robertson et al., 2013). Avoiding the combination of high external inputs with low resource use efficiency remains a major concern for the sustainability of N in agroecosystems (Spiertz, 2010).

Nitrification is the biological oxidation of ammonia (NH_3_) or ammonium (${\text{NH}}_{4}^{+}$) to oxidized N in the form of nitrite (${\text{NO}}_{2}^{-}$) and further to nitrate (${\text{NO}}_{3}^{-}$). In the majority of agricultural soils, ${\text{NH}}_{4}^{+}$ is rapidly converted to ${\text{NO}}_{3}^{-}$, which may accumulate in the soil solution to high concentrations. Conversion of the cation, ${\text{NH}}_{4}^{+}$, to an anion (${\text{NO}}_{2}^{-}$ or ${\text{NO}}_{3}^{-}$) determines the movement of N through the generally negatively charged soil matrix and therefore strongly influences the fate of N in the soil. Nitrate is more likely than ${\text{NH}}_{4}^{+}$ to move rapidly via mass flow to plant roots, leach out of the root zone or be lost from the soil by denitrification. For these reasons, it is often desirable to manage agricultural soils to reduce nitrification, improve the match between available N supply and plant demand and increase N fertilizer use efficiency.

In classical autotrophic nitrification, the conversion of N takes place in two steps: in the ammonia oxidizing bacteria (AOB) such as *Nitrosomonas* or *Nitrosospira*, ${\text{NH}}_{4}^{+}$ is converted to hydroxylamine and then to ${\text{NO}}_{2}^{-}$ with a net outcome shown in Equation 1. Ammonia oxidizing archaea (AOA) such as *Nitrososphaera* have been shown to oxidize ${\text{NH}}_{4}^{+}$ to ${\text{NO}}_{2}^{-}$ (Schleper and Nicol, 2010) although by a significantly distinct metabolism from the AOB (Kozlowski et al., 2016).

(1) $${\text{NH}}_{4}^{+}+1.5{\text{O}}_{2}\to {\text{NO}}_{2}^{-}+{\text{H}}_{2}\text{O}+2{\text{H}}^{+}$$

While the nitrite oxidizing bacteria (NOB) such as *Nitrobacter* or *Nitrospira* convert ${\text{NO}}_{2}^{-}$ to ${\text{NO}}_{3}^{-}$

(2) $${\text{NO}}_{2}^{-}+0.5{\text{O}}_{2}\to {\text{NO}}_{3}^{-}$$

Recently certain *Nitrospira* bacteria have been found that mediate the entire reaction from ${\text{NH}}_{4}^{+}$ to ${\text{NO}}_{3}^{-}$ within one organism in the Complete Ammonia **Ox**idation to nitrate known as Comammox (Daims et al., 2015; van Kessel et al., 2015).

(3) $${\text{NH}}_{\text{4}}^{+}+{\text{2O}}_{\text{2}}\to {\text{NO}}_{\text{3}}^{-}+{\text{H}}_{\text{2}}\text{O}+{\text{2H}}^{+}$$

Many AOB, AOA, NOB, and Comammox organisms gain energy from these reactions and then grow by the fixation of inorganic C into biomass and are therefore chemolithoautotrophs. While it is convenient to group and discuss organisms by the reactions that they mediate, our recent insights into the complexity and versatility of microbial metabolic modules catalyzing N transformations reminds us that this operational approach is not a static classification (Kuypers et al., 2018), the capability of organisms in their environment is the result of complex genetic potential shaped through their environmental and evolutionary past.

Chemolithotrophic bacteria and archaea that are energetically dependent on oxidizing ${\text{NH}}_{4}^{+}$/NH_3_ and/or ${\text{NO}}_{2}^{-}$ for their growth mediate the majority of nitrification in agricultural soils. For this reason the rate and extent of the nitrification process is closely linked to the abundance and functional ecotypes of these responsible organisms. Simulation modeling of nitrification processes may be improved by inclusion of microbial community or functional gene abundance data into predictive models (Bouskill et al., 2012; Graham et al., 2016; Le Roux et al., 2016; Breuillin-Sessoms et al., 2017). This review covers the main factors controlling nitrification rates in agricultural soils, agricultural practices that may reduce nitrification and associated fertilizer N loss and the potential interactions of nitrification rates and extent with climate change under agricultural management.

## Controls on Nitrification in Agricultural Soils

The main factors controlling the rates of nitrification in agricultural soils include the substrate supply, environmental conditions, organismal populations of nitrifiers and competitors, and the presence of nitrification inhibitors. These factors include those that act directly at the cell level and many that act indirectly affecting the soil habitat of the nitrifying organisms. The timescale for these factors spans from immediate change in rates at minutes to hours spanning to years and decades for changes in the soil organic matter pools and their turnover. There are significant interactions and feedbacks between controlling factors since the populations of active nitrifying microbes is determined by the substrates driving their metabolism and growth. Several mechanistic models simulate nitrification at various levels of complexity and these are compared for their treatment of some of these main controlling factors in Table 1.

**Table 1:** Simulation models including nitrification rate and their treatment of controlling factors.

	**DayCent** (Parton et al., 2001; Del Grosso et al., 2009, 2016; Abdalla et al., 2010)	**DNDC** (DeNitrification DeComposition) (Li et al., 1992, 2012; Giltrap et al., 2010; Gilhespy et al., 2014)	**Ecosys** (Grant, 1994, 2001, 2014; Grant and Pattey, 2003; Grant et al., 2006)	**MicroTrait–N** (Bouskill et al., 2012)
**PARAMETER**				
Nitrification rate	Nitrification rate is a function of ${\text{NH}}_{4}^{+}$, water content, temperature, pH, and texture	rate is a first order function of ${\text{NH}}_{4}^{+}$ concentration, nitrifier biomass, a temperature reduction factor, and a moisture reduction factor	substrate (NH_3_) oxidation under non-limiting O_2_ is calculated from active biomass and from NH_3_ and CO_2_ concentrations (same for ${\text{NO}}_{2}^{-}$)	Briggs Haldane kinetics for ammonia and oxygen for AO and for nitrite and oxygen for NO
Soil ammonia/ammonium	Model derived soil ammonium × maximum fraction nitrified	${\text{NH}}_{4}^{+}$ concentration used in Michaelis-Menten kinetics	Solution ${\text{NH}}_{4}^{+}$/NH_3_ drives rates	Dynamic solution NH_3_ driven by pH and consumption
Mineralization	Net mineralization fraction (.20)	Submodel of decomposition	Submodel of decomposition	Inputs but not linked
Nitrite	Not modeled	Not modeled	Modeled explicitedly	Product of AO
Oxygen in soil	Limited at high WFPS, soil physical properties control gas diffusivity and 0_2_ demand	DOC Anaerobic balloon concept	Consumption by microbial groups, O_2_ uptake in competition with heterotrophs, roots; then diffusion to nitrifier	O_2_ use by nitrification reactions
Temperature	Ts estimated based on heat flux and soil heat capacity, used as T factor	Ts estimated based on heat flux and heat flow used as a T factor compared to optimum	Uses modeled *T_*s*_* applied through Arrhenius function	Different temperature optima across guilds Optimum set to 25°C
Water	Optimum WFPS about 55% if low scales down nitrification from moisture stress, high scaled down by DOC	Soil moisture content converted to WFPS, Moisture reduction factor, optimum at 90% WFPS	Water film thickness from modeled water potential	Assumed in water films
Nitrifier abundance	Not modeled	Nitrifier biomass, Nitrifier-bacterial growth and death rate are functions of DOC and a T factor.	(Active) Nitrifier biomass growth by double Monod functions of CO_2_s and NH_3_s AO and NO separately	Growth and death of biomass through C and N equations
Nitrifier denitrification (N gas from nitrification)	Fraction of N nitrified	Function of water-filled pore space and quantity of N nitrified	Process included when O_2_ limits rate of NH_3_ oxidation	Decomposition of hydroxylamine or detoxification of ${\text{NO}}_{2}^{-}$ due to uncoupling

*AO, ammonia oxidation; NO, nitrite oxidation; T, temperature; s, soil; WFPS, water filled pore space; DOC, dissolved oxygen concentration*.

### Substrate Supply Effects on Nitrification

The substrate supply for energy yielding reactions (Equations 1–3) are important factors controlling nitrification in agricultural soils. The availability of ammonia/ammonium (${\text{NH}}_{4}^{+}$/NH_3_), ${\text{NO}}_{2}^{-}$, and O_2_ often limits both the rate of nitrification and the size of the resultant nitrifier populations (Grant, 1994; Bouskill et al., 2012; Nowka et al., 2015; Venterea et al., 2015; Ouyang et al., 2018). Although O_2_ is an important substrate for nitrification; its availability is closely linked to soil water status and thus O_2_ availability will be discussed with environmental factors below. In agricultural soil environments, the substrate pool of ${\text{NH}}_{4}^{+}$/NH_3_ is increased by (1) additions of urea and ammonical fertilizers, (2) deposition of animal wastes (urine and feces), (3) atmospheric deposition of ${\text{NH}}_{4}^{+}$, (4) biological N fixation, and (5) ${\text{NH}}_{4}^{+}$ production via mineralization. The competing consumptive processes including microbial assimilation (immobilization), plant assimilation, and ammonia volatilization decrease available ${\text{NH}}_{4}^{+}$/NH_3_ (Figure 1).

Nitrification rates are often modeled as first-order with respect to ${\text{NH}}_{4}^{+}$/NH_3_ pools (appropriate for lower concentrations) or using Michaelis-Menten equations (Norton and Stark, 2011; Bouskill et al., 2012; Inselsbacher et al., 2013; Breuillin-Sessoms et al., 2017). Often ammonia oxidation rates are assumed to limit the overall rate of nitrification and nitrite does not accumulate. Some important exceptions are described below. The substrate for the crucial integral membrane protein ammonia monooxygenase (AMO) is generally accepted as solution NH_3_ (Suzuki et al., 1974). All known substrates and competitive inhibitors of AMO are non-polar (Suzuki et al., 1974; Hooper et al., 1997; Arp et al., 2002) suggesting that the AMO active site is a non-polar environment. Rapid equilibration in aqueous environments means this solution NH_3_ form is transient and seldom directly measured in soil environments. The determination of solution ${\text{NH}}_{4}^{+}$/NH_3_ in soils is complicated by the microsite variability in pH and the sorption capacity of the soil (Venterea et al., 2015). These relationships are of particular importance after fertilization or urine deposition resulting in localized high concentrations of substrates. Many but not all AOB and AOA are capable of using urea and have genes encoding urease enzymes and urea transporters (De Boer and Laanbroek, 1989; Burton and Prosser, 2001; Koper et al., 2004; Tourna et al., 2011; Lu and Jia, 2013; Shen et al., 2013). Some comammox organisms and NOB also possess ureolytic activity (Koch et al., 2015, 2019; Palomo et al., 2018).

The injection of anhydrous ammonia and banding of urea fertilizers in soils results in temporarily extremely high ${\text{NH}}_{4}^{+}$/NH_3_ concentrations and high pH as well. In these localized zones total ${\text{NH}}_{4}^{+}$/NH_3_ may reach from several hundred up to 2,000 mg N/kg soil (Venterea et al., 2015). Under these episodic high concentrations, existing populations of ammonia oxidizers are operating at maximum capacity or even inhibited by high substrate (NH_3_) or product (${\text{NO}}_{2}^{-}$) concentrations.

In general, ${\text{NO}}_{2}^{-}$ does not accumulate in soils except under transient conditions that have decreased the population or inhibited the activity of nitrite oxidizers. The intensive application of ammonical fertilizers (i.e., urea or anhydrous NH_3_) may result in ${\text{NO}}_{2}^{-}$ accumulation due to the inhibition of ${\text{NO}}_{2}^{-}$ oxidation from the toxicity of high NH_3_ levels in the application zone (Schmidt, 1982; Maharjan and Venterea, 2013; Giguere et al., 2017) or from subsequent localized lowering of pH and production of nitrous acid (Venterea and Rolston, 2000a). Any circumstance under which the rate of NH_3_ oxidation exceeds that of ${\text{NO}}_{2}^{-}$ oxidation will result in accumulation. This accumulation of ${\text{NO}}_{2}^{-}$ is an important driver of N_2_O/NO_x_ production by both biological and abiotic reactions (Venterea et al., 2015; Heil et al., 2016; Breuillin-Sessoms et al., 2017; Giguere et al., 2017). The interaction of soil pH, buffering capacity and ionization of NH_3_ and ${\text{NO}}_{2}^{-}$ may be useful predictors of ${\text{NO}}_{2}^{-}$ accumulation and the associated increased production of N_2_O/NO_x_ via nitrification and nitrifier-denitrification (Venterea and Rolston, 2000b).

The deposition or application of animal wastes due to grazing or amendments leads to local zones of high organic N, urea and ${\text{NH}}_{4}^{+}$/NH_3_. Typically, over 70% of the N in ruminant urine is found as urea and localized deposition zones reach ${\text{NH}}_{4}^{+}$/NH_3_ concentrations and elevated pH similar to those found in urea fertilizer bands. For intensively grazed pastures levels of deposition may reach up to 600–1,200 kg N ha^−1^ significantly exceeding uptake by pasture plants (Hamonts et al., 2013). Applications of manures and composts to agricultural lands adds urea, organic N and ${\text{NH}}_{4}^{+}$/NH_3_ often stimulating nitrification rates in the receiving soils (Li et al., 2012).

Rates of NH_3_ emissions are increasing with agricultural activities accounting for 80–90% of anthropogenic emissions. Increasing manure production and N fertilizer use drives NH_3_ emissions and then subsequent deposition to land surfaces both globally and locally. Total N in wet and dry deposition approximately tripled during the last century (Simkin et al., 2016). Deposition typically occurs at a sustained elevated level in contrast to the large pulses of ${\text{NH}}_{4}^{+}$/NH_3_ due to fertilization. These increased inputs can be expected to affect soil inorganic N pools for surface soils, most importantly in low fertility ecosystems.

Rates of ${\text{NH}}_{4}^{+}$/NH_3_ production and consumption are important controls on the rate and extent of nitrification (Norton, 2008; Grant et al., 2016). Mineralization is the general term for the conversion of organic N to inorganic N as either ${\text{NH}}_{4}^{+}$ or further to ${\text{NO}}_{2}^{-}$/${\text{NO}}_{3}^{-}$, ammonification is the conversion of organic N to the ${\text{NH}}_{4}^{+}$ form while immobilization is the assimilation of inorganic N to organic N generally mediated by microorganisms. Mineralization-immobilization turnover (MIT) refers to the combined transformations between organic and inorganic N that accompanies the growth and death of the soil biota. The supply of ${\text{NH}}_{4}^{+}$ for nitrification depends upon the balance of mineralization to immobilization and the quality and quantity of substrate for decomposition. Soil organic C and N pool size are effective predictors of soil mineralization rates when considered over continental scales (Booth et al., 2005). In tightly coupled N cycles the pool size of ${\text{NH}}_{4}^{+}$does not reflect the supply of this substrate. Plant uptake may compete directly for ${\text{NH}}_{4}^{+}$. Assessment of the true inorganic N supplying capacity of the soil, i.e., gross ammonification, may better represent the absolute flux of inorganic N produced by soil N mineralization (Van Groenigen et al., 2015). The fraction of the mineralized N that is nitrified or the ratio of gross nitrification to mineralization (GNR/GMR) (Table 2) is considered an index of the nitrifying capacity of soils (Booth et al., 2005; Habteselassie et al., 2006). Nitrification potentials that measure short-term nitrite/nitrate production in shaken soil slurries with non-limiting substrate supply, are useful indicators of the enzymatic potential for nitrification but are not necessarily predictive of *in-situ* rates (Hart et al., 1994; Norton and Stark, 2011). Soils that have received repeated applications of composts and manures typically show increases in the ratio of gross nitrification rate to nitrification potential (GNR/NP) because high rates of mineralization continuously supply substrate ${\text{NH}}_{4}^{+}$ (Table 2) (Habteselassie et al., 2006; Ouyang et al., 2016). Relationships of mineralization to nitrification rates are best assessed through the determination of gross rates using isotope pool dilution and modeling approaches. These comparisons of gross and net nitrification rates are evidence that net nitrification measurements are poor predictors of gross nitrification rates for many soils (Stark and Hart, 1997; Burger and Jackson, 2003, 2004; Habteselassie et al., 2006; Norton and Stark, 2011; Han et al., 2012).

**Table 2:** Ratios of gross and net N transformation rates for an agricultural soil under silage corn that received ammonium sulfate (AS), dairy waste compost (DC), and dairy liquid waste (LW) at 100 and 200 kg available N ha^−1^ for 6 years.

**Treatment**	**NNR^a^/GNR**	**GNR/GMR**	**GNR/NP**
AS100	0.38^ba^	0.59^b^	0.10^b^
AS200	0.36^ba^	0.66^b^	0.09^b^
DC100	0.16^b^	1.36^ab^	0.58^a^
DC200	0.15^b^	1.88^a^	0.64^a^
LW100	0.46^ab^	0.78^ba^	0.18^b^
LW200	0.69^a^	0.66^b^	0.22b^a^

*GNR/GMR, and GNR/NP values are means for year 1999 to 2002 (Habteselassie et al., 2006). Numbers followed by same letter within a column are not significantly different at P < 0.05*.

a *From laboratory incubation measurements*.

*NNR, net nitrification rate; GNR, gross nitrification rate; GMR, gross mineralization rate; NP, nitrification potential*.

### Environmental Conditions–Temperature, Soil Moisture, Aeration, and pH

#### Temperature

**Figure 2:**
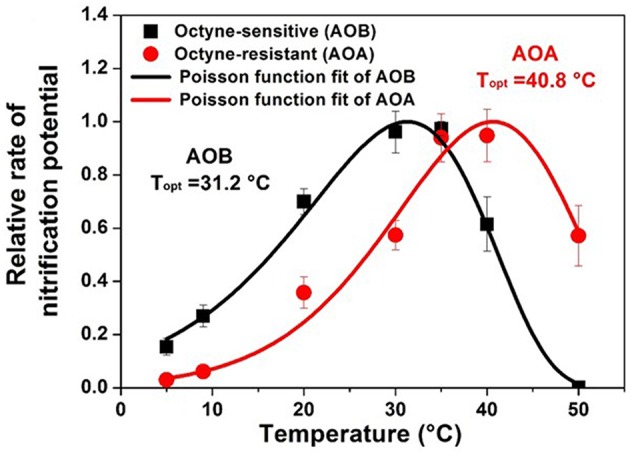
Temperature response of the relative potential nitrification rates for AOB (octyne-sensitive) and AOA (octyne-resistant) from a calcareous agricultural soil in Utah, USA. Rates are normalized to the fraction of maximum nitrification potentials at optimum temperature. Lines predicted by generalized Poisson density equation (Adapted from Ouyang et al., 2017).

The response of nitrification to temperature has been evaluated in a diverse range of soils, and the optimum temperature for nitrification has been found to be environment specific (Stark, 1996; Parton et al., 2001; Lu et al., 2018). Across a range of North American ecosystems, the community composition of AOB was correlated with temperature as indicated by mean annual temperature (Fierer et al., 2009). The temperature optimum for nitrification in an AOA dominated soil has also been found to be increased under selective warming pressure and to have selected for temperature optima related to the environment (Daebeler et al., 2017). Overall soil microbial communities tend to be temperature generalists since they are adapted to wide swings of temperature in surface soil habitats (Wallenstein and Hall, 2012). Generally, the optimum temperature for maximum short-term nitrification rates (i.e., V_max_) may exceed the temperatures normally experienced at the site under consideration and may exceed the temperature optimum for growth of nitrifier biomass (Stark and Firestone, 1996; Taylor et al., 2017). Cultured AOB from soils generally have temperature optimum between 25 and 30°C (Jiang and Bakken, 1999), but *N. cryotolerans* from the Arctic Ocean has a temperature optimum for growth of 22°C and can grow at 0°C (Koops et al., 1991). There is evidence for soil nitrifier activity under similarly cold temperatures typical of winter season soils (2–10°C) (Cookson et al., 2002) and for nitrification in AOA dominated Artic soils (Alves et al., 2013). Recent evidence suggests that certain groups of acid tolerant AOA may also be adapted to lower temperatures regimes (Gubry-Rangin et al., 2017). The temperature response of nitrification has been modeled using the Arrhenius equation (Grant, 1994), a Poisson density function (Stark, 1996; Ouyang et al., 2017), square root (SQRT) function or using macromolecular rate theory (MMRT) (Taylor et al., 2017). Studies performed with pure cultures and with mixed environmental consortia from temperate agricultural soils consistently indicate that AOA activity has a higher temperature optima and higher temperature minimum than AOB activity (Figure 2) (Ouyang et al., 2017; Taylor et al., 2017; Lu et al., 2018). Modeled temperature response parameters may be useful for trait based modeling linking microbial populations to nitrification rates (Bouskill et al., 2012; Breuillin-Sessoms et al., 2017).

#### Moisture/Aeration

Soil moisture affects nitrification rates through several confounding influences of substrate availability of both ammonium and oxygen by diffusion and direct effects of dehydration at very low water potentials. These interdependent factors often confound experiments to determine the role of soil drying and wetting on nitrification rates under field conditions (Stark and Firestone, 1995; Placella and Firestone, 2013). Optimum water filled pore space (WFPS) for nitrification is around 55% for fine textured soils and around 40% WFPS for coarse textured soils (Parton et al., 2001) see Supplemental Figure 1). Nitrification in soils saturated with water (i.e., water potential approaching 0 kPa) is inhibited due to lack of available oxygen. Nitrification nearly halts in very dry soils (< -3.0 MPa), such as found under seasonal dry xeric or aridic soil climates. In general, the diffusion of substrates limits nitrification activity most near optimum water potentials, whereas the adverse physiologic effects associated with cell dehydration will be the most limiting factor at very low water potentials (Stark and Firestone, 1995). In the nitrification submodel of DayCent (Del Grosso et al., 2012) nitrification is limited by moisture stress when soil water-filled pore space (WFPS) is too low and by O_2_ availability when WFPS is high based on soil textural class (Supplemental Figure 1). In the highly detailed model Ecosys, O_2_ availability is based on water film thickness and the wide range of competing microbial processes consuming O_2_ (Grant and Pattey, 2003).

#### Soil pH

The soil pH is one of the most important factors controlling rates and product accumulation from nitrification see Supplemental Figure 1 (Parton et al., 2001; Kyveryga et al., 2004). Rates of both ammonia and nitrite oxidation are generally favored by neutral to slightly alkaline soils and it is in these soils that the largest losses or accumulations of ${\text{NO}}_{3}^{-}$ generally occur. Management of agricultural soil pH by liming is common practice in vast areas of crop production and is often necessary to offset acidification due to fertilizers. Currently ~40% of the world's arable soils are acidic and this area has recently been increasing (Kunhikrishnan et al., 2016). High rates of nitrification and leaching of ${\text{NO}}_{3}^{-}$ further acidify agricultural soils (Schroder et al., 2011). During the Twentieth century, observations that nitrification was occurring in acids soils from both natural and agricultural ecosystems continued to accumulate with observations from tea plantations, heath soils and coniferous forests (De Boer and Kowalchuk, 2001). During this same time frame the available isolates of AOB were fairly intolerant of acidity and their nitrification rates decreased dramatically as pH decreased (De Boer and Kowalchuk, 2001). These observations were partially explained by the known low concentrations of NH_3_ (${\text{NH}}_{4}^{+}$/NH_3_ couple has a pK_a_ = 9.25) and the contention that NH_3_ is the actual substrate for ammonia oxidizers (Suzuki et al., 1974). The use of urea as a substrate, microsite variability of soil pH and heterotrophic nitrification were able to explain some portion of nitrification observed in acid soils (Burton and Prosser, 2001). Since the role of AOA in ammonia oxidation in the soil environment was revealed (Treusch et al., 2005; Leininger et al., 2006; Nicol et al., 2008) the importance of AOA in the ammonia oxidation of acid soils has gained increasing support (Nicol et al., 2008; Gubry-Rangin et al., 2010; Yao et al., 2011, 2013; Prosser and Nicol, 2012; Li et al., 2018). Members of the AOA *Nitrosotalea* lineage are abundant and widely distributed in acidic soils globally (Gubry-Rangin et al., 2011). An obligate acidophilic isolate, Ca. *Nitrosotalea devanaterra*, is unable to grow at neutral pH (Lehtovirta-Morley et al., 2011) and exhibits specialized genomic inventory for functioning under acid conditions (Lehtovirta-Morley et al., 2016b). Soil pH has also been observed to affect the nitrite oxidizer community (Han et al., 2017).

### Effects of Abundance and Community Structure of Nitrifiers on Rates

**Figure 3:**
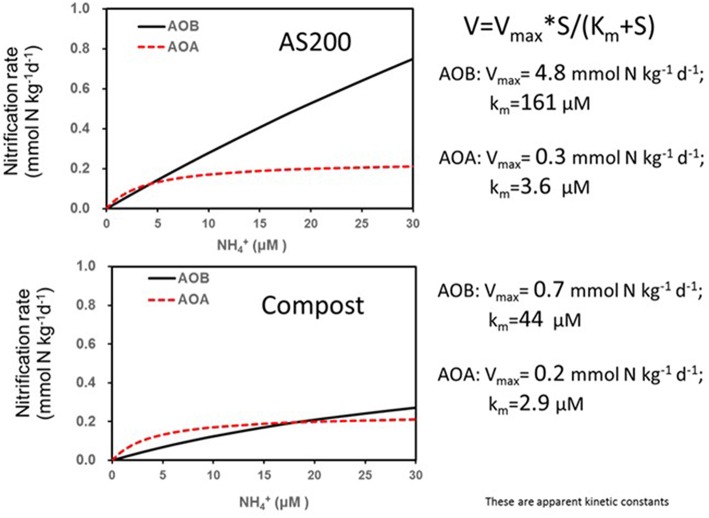
Nitrification rate kinetic models based on substrate concentrations for a calcareous agricultural soil from Utah treated for 3 years with either ammonium sulfate or steer waste compost at 200 kg N/ha. Soils were sampled 28 days after fertilization (adapted from Ouyang et al., 2017).

**Figure 4:**
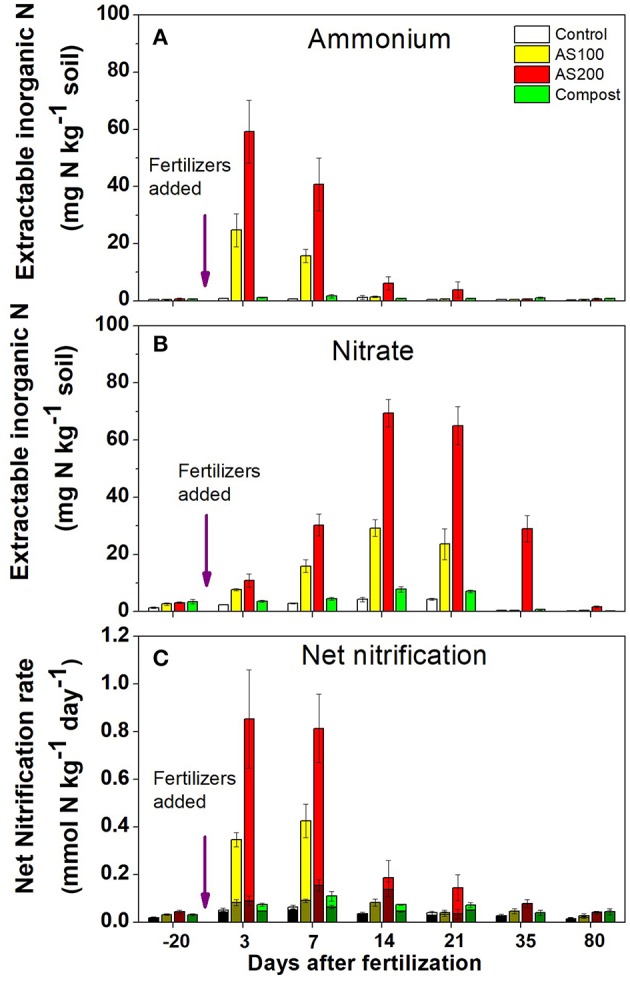
Inorganic N pools and net nitrification rates for a calcareous agricultural soil from Utah. Field plots had been treated for four previous years with either no N fertilizer (control), ammonium sulfate 100 kgN/ha (AS 100), ammonium sulfate 200 kgN/ha (AS 200) or steer waste compost at 200 kg N/ha (Compost) under silage corn production. Observations for 2015 growing season for: **(A)** ammonium N pool size, **(B)** nitrite +nitrate N pool size, **(C)** net nitrification rate for octyne-sensitive (AOB) net nitrification, octyne-resistant (AOA) net nitrification, and total net nitrification. Octyne-resistant nitrification rates (AOA) are the shaded bottom portion of each bar, octyne-sensitive nitrification rates (AOB) are the lighter top portion of each bar (Adapted from Ouyang et al., 2017).

There is a complex interaction between the soil environment, plant community and management (especially fertilization) that determines the community structure of nitrifiers in agricultural soils (Bertagnolli et al., 2016; Han et al., 2018). The abundance and ecotypes of the ammonia and nitrite oxidizers present in the soil may control the immediate rate of nitrification especially when substrate is in excess. Generally, when fertilizers are applied the existing populations respond relatively quickly to the transient increased substrate availability dependent upon favorable environmental conditions. Comparisons between the responses of AOB and AOA to fertilizers suggest that the kinetics of their responses to substrate are distinct (Prosser and Nicol, 2012). An example from Utah agricultural soil comparing the AOB and AOA response shows that AOA reached a lower Vmax at a much lower substrate availability (Figure 3) (Ouyang et al., 2017). Nitrification driven by AOA was also found to be saturated at relatively low ${\text{NH}}_{4}^{+}$ in a range of Oregon soils (Giguere et al., 2015). These observations explain why some studies have observed a positive correlations between the abundance of AOB and nitrification potential rates performed at relatively high ${\text{NH}}_{4}^{+}$ (1 mM) but little or no correlation with potential rates and AOA abundances (Jia and Conrad, 2009; Taylor et al., 2012; Ouyang et al., 2016). In contrast to these observations, under conditions such as acid soils that favor AOA, nitrification rate is often proportional to AOA gene abundance (Yao et al., 2011). In Scotland, a survey that included nitrification potentials, AOB and AOA abundances and their communities found that specific phylotypes of AOA and AOB were linked to soil niches described by combinations of soil pH and fertilization (Yao et al., 2013). In general, acidic soils from non-fertilized systems exhibited lower rates and were dominated by distinctive AOA phylotypes. In calcareous agricultural soils from Utah, nitrification potentials (at 1 mM ${\text{NH}}_{4}^{+}$) were dominated by activity of AOB related to *Nitrosospira* even though the AOA were more abundant. However, in these same soils, net and gross nitrification rates were mediated by AOB in the first weeks following fertilization then, after ammonium was depleted, the activity was dominated by AOA Figure 4 (Ouyang et al., 2017). Candidatus *Nitrosocosmicus franklandus* is an AOA strain (*Archaea, Thaumarchaeota, Nitrososphaerales*) isolated from circum-neutral pH, fertilized soil in Scotland (Lehtovirta-Morley et al., 2016a) has an overlapping ammonia tolerance to known AOB soil isolates. In the acidic red soils of China after 16 years of contrasting fertilizer treatments (He et al., 2007), the AOA remained dominant but both AOA and AOB abundances were increased by organic and inorganic fertilizers, both AOA and AOB played a role in nitrification activity. The abundance of AOA and AOB has been suggested as a bioindicator for soil monitoring based on their differential responses to soil management and relative ease of quantification by real-time PCR (Wessen et al., 2010; Wessén and Hallin, 2011).

Enrichment and pure culture studies of the AOB suggest that substrate kinetics and growth response of AOB differ even within a genus (Jiang and Bakken, 1999; Koops and Pommerening-Roser, 2001; Bollmann et al., 2002; Webster et al., 2005; Bouskill et al., 2012; Sedlacek et al., 2019). Different ecophysiology within the AOA is also indicated by pure culture work (Gubry-Rangin et al., 2011; Martens-Habbena and Stahl, 2011; Hatzenpichler, 2012; Kits et al., 2017).

Fewer assessments have been made of the NOB communities of agricultural soils. Several studies suggest nitrite oxidation in agricultural soils is primarily catalyzed by NOB communities with members from *Nitrospira* and *Nitrobacter* (Freitag et al., 2005; Xia et al., 2011; Pester et al., 2014). Higher potential rates of nitrite oxidation have been found to be associated with *Nitrobacter* vs. *Nitrospira* dominated communities and shifts toward *Nitrobacter* types are often associated with changes in management such as nitrogen fertilization and tillage (Attard et al., 2010; Le Roux et al., 2016; Han et al., 2018). The growth of *Nitrobacter* populations as indicated by *nxrA* gene copies has been associated with rapid nitrite use and lowered N_2_O emissions (Venterea et al., 2015). *Nitrobacter vulgaris* was also found to quickly decrease accumulated nitrite and prevent N_2_O emissions in several Oregon soils (Giguere et al., 2017). Pure cultures of *Nitrospira* spp. generally exhibit higher affinities and lower Vmax than *Nitrobacter spp*. (Nowka et al., 2015). Kinetics of the comammox bacterium, *Nitrospira inopinata*, suggest an oligotrophic lifestyle as well (Kits et al., 2017).

Feedback between fertilizer application and abundance occurs because both the activity and the abundance of nitrifying organisms increase following fertilization with ammonical N fertilizers (He et al., 2007; Ouyang et al., 2016, 2018; Xiang et al., 2017; Orellana et al., 2018). A recent meta-analysis examined the impact of N fertilization on the abundance of N cycling genes in agricultural soils showed that the positive effect size was significant for both the AOA and AOB from a survey of ~100 samples each (Ouyang et al., 2018). In an earlier meta-analysis (Carey et al., 2016) the AOB abundance was found to be more responsive to N fertilization than that of AOA for the majority of observations. AOB abundance was associated with increased nitrification potentials in fertilized soils. In Utah soils, both the abundance and the community of ammonia AOB were more responsive than those of AOA to repeated annual applications of ammonium sulfate fertilizer (Ouyang et al., 2016). Generally, nitrification potential activities were more sensitive to agricultural management practices and environment disturbance than to the abundance and diversity of nitrifiers. For example, in our Utah soil, nitrification potentials were significantly increased by N fertilizers after the first fertilization, while *amoA* gene abundance and diversity showed no significant difference among treatments (Ouyang et al., 2016). Similarly, we found that the nitrite oxidation potentials were significantly stimulated by fertilizers while *nxrB* abundances were not affected (Ouyang, 2016). These *amoA* and *nxrB* gene measurement were based on soil DNA, while the rate of ammonia oxidation may be more related to the relationship among transcription, translation, and enzyme function (Nicol et al., 2008; Myrold et al., 2014; Rocca et al., 2015).

Regulation of transcription of nitrification related genes has been examined both in pure cultures (Sayavedra-Soto et al., 1998, 2015; Bollmann et al., 2005; Hawkins et al., 2007; Starkenburg et al., 2008; Park and Ely, 2009; Radniecki and Lauchnor, 2011), and in soil or sediment environments (Tourna et al., 2008; Di et al., 2010; Gubry-Rangin et al., 2010; Abell et al., 2011; Herrmann et al., 2012; Placella and Firestone, 2013) primarily targeting *amoA* transcription. In a meta-analysis of functional genes and transcript abundance and their relationship to process rates (Rocca et al., 2015) there was less correlation between transcript level (mRNA) and process rates than with gene abundance and process rate. This lack of relationship between transcription and process rate is not surprising considering differences in transcript stability (turnover), transient and episodic rates of transcription and subsequent translation and difficulties with methods for determining transcript abundance in environmental matrices.

A proteomic approach might be appropriate for explaining short-term changes in nitrification activity. The ideal method is to extract and purify key enzymes such as AMO and NXR directly for assays in soils, but the membrane-bound feature of these enzymes makes this strategy difficult (Arp et al., 2002; Kerou et al., 2016). However, the recent study on activity-based protein profiling of AMO in *Nitrosomonas europaea* may pave a way to indirectly quantifying active AMO fluorescently in soils (Bennett et al., 2016). Nitrification is likely the soil N cycle process for which we are approaching a level of understanding when we may include some nitrifier community characteristics into process models using trait-based modeling approaches (Bouskill et al., 2012; Le Roux et al., 2016).

### Plant and Microbial Interaction With Nitrifiers

Plants take up and assimilate both ${\text{NH}}_{4}^{+}$ and ${\text{NO}}_{3}^{-}$, but often shows substantial differences in preference for one inorganic N form (Marschner, 2011). This ${\text{NH}}_{4}^{+}$ or ${\text{NO}}_{3}^{-}$ preference of plant species could exert differential effects on nitrifiers (Patra et al., 2006; Skiba et al., 2011; Thion et al., 2016). Plant often stimulates soil N transformation processes by releasing C into the rhizosphere either as root exudates or as direct transfers to mycorrhizal fungi (Phillips et al., 2011; Shi et al., 2016; Meier et al., 2017). A meta-analysis summarized that N transformation processes were significant higher in rhizosphere than bulk soil, including net and gross N mineralization and net nitrification (Finzi et al., 2015). Rhizosphere interactions have been observed to decrease nitrification (net and gross rates) by favoring plant and microbial assimilation of ${\text{NH}}_{4}^{+}$(Hawkes et al., 2007). Some plants are able to produce nitrification inhibitors in their root exudates, and therefore suppress nitrifier activities (Subbarao et al., 2013, 2015; Coskun et al., 2017). While competition for N between plants and microbes is very strong in the rhizosphere, it is not clear if nitrifiers will outcompete heterotrophic microbes in the rhizosphere (Kuzyakov and Xu, 2013).

Evidence from pure cultures indicate that *Nitrosomonas* spp. are weak competitors for ${\text{NH}}_{4}^{+}$, compared to heterotrophic bacteria (Verhagen and Laanbroek, 1991; Verhagen et al., 1994; Bollmann et al., 2002). In many agricultural soils, gross nitrification rates are often 1–75 fold higher than rates of microbial ${\text{NH}}_{4}^{+}$ assimilation indicating that soil nitrifiers are strong competitors for ${\text{NH}}_{4}^{+}$ (Burger and Jackson, 2003; Booth et al., 2005; Inselsbacher et al., 2010). Heterotrophic microbes may assimilate nitrate as well especially under high organic matter and high C availability. The balance between organic C and ${\text{NH}}_{4}^{+}$ availability will likely determine the fate of ${\text{NH}}_{4}^{+}$ during competition in agricultural soils.

Arbuscular mycorrhizal fungi (AMF) may play an important role in mediating availability of ${\text{NH}}_{4}^{+}$ to nitrifiers. AMF could directly compete for ${\text{NH}}_{4}^{+}$ (Veresoglou et al., 2011, 2012; Chen et al., 2013; Storer et al., 2018), but also likely exert indirect influences on nitrifiers via the plant (Chen et al., 2013; Veresoglou et al., 2018). AOA community composition was altered more than the AOB community by AMF (Chen et al., 2013). Ectomycorrhizal fungi produce many extracellular enzymes for N mineralization and may increase the availability of soil N (Courty et al., 2010). Interestingly, ectomycorrhizal fungi inoculation changed AOA, but not AOB communities in an acid soil (Li et al., 2019).

The biological interaction between soil microfauna and microorganisms in the soil food web also mediates soil N cycling (Xiao et al., 2010; Jiang et al., 2014; Trap et al., 2016; Zhu et al., 2018). For example, Xiao et al. (2010) found the presence of bacterivorous nematodes significantly stimulated nitrification activity and changed the community composition of AOB. Interestingly, Zhu et al. (2018) showed bacterivorous nematodes significantly reduced the abundance of AOB, but increased AOA, irrespective of the nematode species in the soil. There may also be a role for bacterial predators such as *Micavibrio* that have been observed in wastewater systems to prey upon *Nitrospira* (Dolinšek et al., 2013). The knowledge of potential environmental interactions between viruses and nitrifiers is limited although genomes of AOB have shown evidence of prophage (Chain et al., 2003; Stein et al., 2007; Norton et al., 2008). More recently prophage induction by stress followed by lysis was demonstrated in *Nitrosospira multiformis* (Choi et al., 2010). The outcome of these complex interactions in agricultural soils is driven by the timing and intensity of organic C and available N and their distribution by mass flow and diffusion through the soil fabric.

## Managing Nitrification in Agricultural Soils

Meeting world food demand while reducing surplus N lost to the environment will require substantial increases in the NUE of agricultural systems (Zhang et al., 2015). Management strategies are needed that minimize the risk of N loss even in high productivity systems that necessarily require high N inputs. As the demand for food production increases globally, the production and use of N fertilizer will likely continue to increase from ~110 Tg N in 2013 up to 120 Tg by 2018 (FAO, 2015). The vast majority of N fertilizers applied to soils are in the ammonical forms including urea (57% in 2013 and increasing) and are therefore subject to nitrification after application. In the United States, ~50% of this N fertilizer is used on maize (corn) crops (USDA, ERS, 2018). As agriculture intensifies, there will be higher levels of N applied to reach the yield potential of the most productive varieties if current conventional management continues. Common principles for N management include the “4Rs” approach of applying the right source, at the right rate, at the right time in the right place (Clarke and Beegle, 2014). Many appropriate technologies are currently available to reduce nitrification, greenhouse gas (GHG) emissions and N losses but these may require appropriate incentives for farmers to adopt (Robertson et al., 2013). Complex models such as DayCent (Del Grosso et al., 2012) that are used for the estimation of the flux of N_2_O from agricultural soils include nitrification submodels. The outcomes of management activities may be simulated and assessed with these tools.

China has some of the most intensive use of N fertilizers and associated high levels of N loss. In a meta-analysis of Chinese agriculture, management practices designed to minimize N loss were assessed including: the application of controlled-release N fertilizers, nitrification inhibitors (NI) and urease inhibitors (UI), higher splitting frequency of fertilizer N application, lower basal N fertilizer (BF) proportion, deep placement of N fertilizer, and optimizing N rate based on soil N test (Xia et al., 2017). These knowledge-based N fertilization practices were generally effective at reducing N loss by leaching, runoff and GHG emission while showing some increases in economic return. Split applications of N and the use of enhanced efficiency fertilizers including those with polymer coatings and urease and nitrification inhibitors will make increased economic sense if they are used selectively under those environmental conditions where the potential N loss is high (Motavalli et al., 2012).

Management practices that improve or maintain soil health such as disturbing the soil less (reduced tillage), growing greater diversity of crops (in rotation and as diverse mixtures of cover crops), maintaining living roots in the soil as much as possible (with crops and cover crops), and keeping the soil covered with residue at all times will increase the resiliency of agroecosystems and decrease N losses (Zhang et al., 2015). These practices will likely result in decreased net nitrification while maintaining yields. The implementation of this knowledge to build more resiliency into our agricultural systems will need support from socioeconomic policy research.

### Management to Control Ammonium Substrate Availability

**Figure 5:**
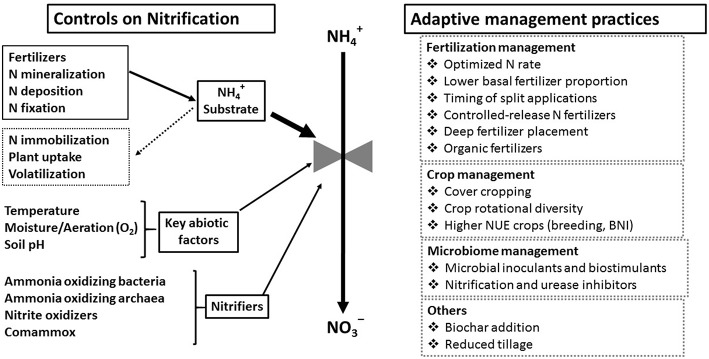
Relationship of controlling factors for nitrification to adaptive management practices promoting systems with higher nutrient use efficiency.

The goal of N fertilizer rate recommendations is to estimate the gap between the N supplied by the soil and the N required for the crop to reach an optimum yield. In the United States and Europe, regional yield response curves and the fertilizer-crop price ratio are often used to provide recommendations to farmers on economically optimal N application rates (Sawyer et al., 2006; Morris et al., 2018). Decreasing this basal N fertilizer rate will logically decrease N availability to nitrification but risks reducing yields enough to be an economic disadvantage and even increase overall environmental impacts. Therefore, rather than decrease overall N rate, approaches designed to improve NUE while maintaining yields may mitigate the risks associated with nitrification. Strategies for controlling ${\text{NH}}_{4}^{+}$ substrate availability include timing of fertilization to coincide with rapid plant uptake, formulation of fertilizers as slow release forms and/or with inhibitors (e.g., urease inhibitors), keeping plants growing continuously to assimilate N, and increasing microbial N immobilization (Figure 5).

#### Timing of Fertilization to Coincide With Rapid Plant Uptake

Generally, N fertilizers can be applied either before planting, as a sidedress, or as a split preplant-sidedress fertilizer treatment. Nitrogen is used more efficiently if applied during the growing season prior to the time of maximum plant uptake rate, as compared to application before the crop is planted (Sawyer et al., 2006). The timing of this split application may be based on crop stage or other plant or soil testing indicators such as the presidedress nitrate test (PSNT). Sidedress fertilization has been observed to reduce yield scaled N_2_O by 60% vs. fall fertilization (Abalos et al., 2016) and often results in improvements in NUE (Ma et al., 2010). Unfortunately, there remain large areas in the US Midwest and Canada where convenience favors anhydrous ammonia application to drier soils during the fall preceding spring planting. This approach is based on the principle that cold soil temperatures will slow nitrification sufficiently to retain fertilizer in the soil. Fall applications typically reduce NUE and must be timed carefully to wait until soil temperatures decrease enough to postpone nitrification activity until spring. Nitrification inhibitors are often combined with fall applications to delay nitrification but these may not remain effective through to the following spring.

#### Keep Plants Growing Continuously to Assimilate N

Competition with plants for available N can decrease nitrification and decrease nitrate accumulation. In many non-agricultural systems, plant N uptake occurs across seasons and N is retained in organic forms and in plant roots. Additionally, even when nitrification is occurring, there may be little net nitrification measurable because of nitrate use by plants and heterotrophic microbes (Stark and Hart, 1997; Norton and Stark, 2011). A range of N conserving mechanisms have evolved in natural ecosystems including direct uptake of organic N by plants (by short-circuiting mineralization) and suppression of nitrification. These mechanisms essentially close the N cycle and facilitate soil organic N accumulation. The use of cover crops, living mulches and catch crops keeps living plant roots in the soil, adds organic matter to the system, and decreases nitrate accumulation and potential leaching (Abdalla et al., 2019). Cover crops must be managed carefully especially in drier climates to avoid decreases in the productivity of the primary crop due to water or nutrient uptake while promoting soil nitrate recycling.

#### Controlled-Slow Release Fertilizers

Slow/controlled release fertilizers are designed to better match the timing of nutrient release to the plant demand. Because of cost factors, their use in agricultural settings is limited although they are widely used in horticultural applications. Urea is one of the most widely used fertilizers in agriculture and is extremely soluble. Slow release coatings may be applied to limit solubility and delay urea hydrolysis and subsequent nitrification. Urea coatings include organic polymer coatings and inorganic coatings such as sulfur, their characteristics and merits of these materials have been reviewed recently (Naz and Sulaiman, 2016).

#### Intensify Soil Internal N Cycling

**Figure 6:**
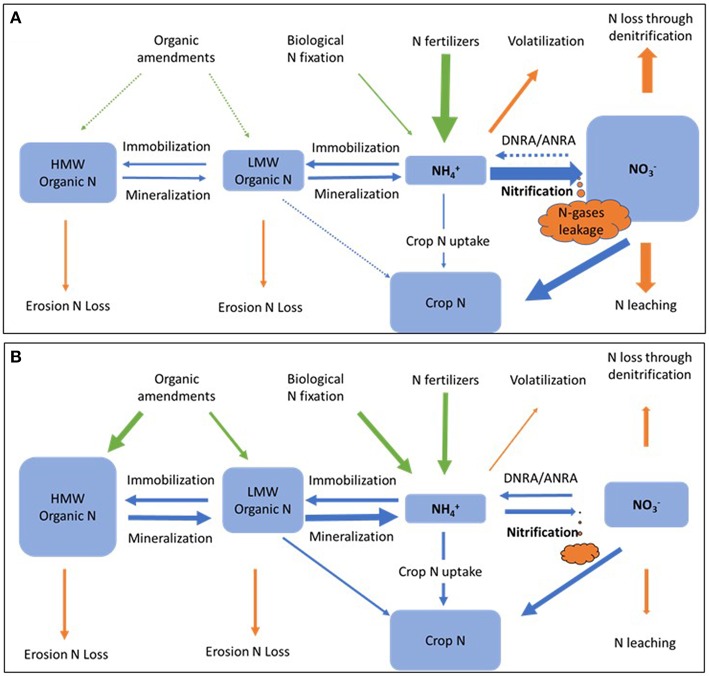
Hypothetic nitrogen pools and flows of high-nitrifying **(A)** and low-nitrifying **(B)** agricultural systems. Arrows represent nitrogen inputs (green), losses (orange), and transformations (blue). HMW, high molecular weight; LMW, low molecular weight; DNRA, dissimilatory nitrate reduction to ammonium; ANRA, assimilatory nitrate reduction to ammonium.

The use of inorganic fertilizers simplifies the soil internal N cycling process, leading to a high-nitrifying agricultural system (Figure 6A). Nitrate is often a dominant N pool, especially shortly after fertilization, in these agricultural soils. When the proportion of N supply to the plant by N fixation and N mineralization is increased relative to fertilizers sources then a low-nitrifying agricultural system is favored that reduces N loss and improves NUE (Figure 6B). Increased diversity of N cycling functional groups may also help retain N in soil. Intensified internal N cycling may be accomplished by the addition of high C organic amendments such as compost, manure, and biochar (Paustian et al., 2016); and by direct inoculation of N-fixation and mineralization promoting bacteria and AMF (Hu and He, 2018).

### Inhibit Nitrifiers Directly

#### Nitrification Inhibitors

Nitrification inhibitors (NIs) slow the microbial conversion of ammonium-N to nitrate-N (nitrification), reducing the risk of loss through leaching or denitrification and thereby increasing the NUE of fertilizers. Many synthetic NIs act on the ammonia monooxygenase enzyme often as competitive suicide subtrates (for example acetylene) (McCarty, 1999). Several nitrification inhibitors that are widely used in agriculture include: (1) 2-chloro-6-(trichloromethyl) pyridine (nitrapyrin), (2) dicyandiamide (DCD), and (3) 3,4-dimethylepyrazole phosphate (DMPP). Urease inhibitors, such as N-(n-butyl) thiophosphoric triamide (NBPT), are used to decrease urea hydrolysis and volatilization. Meta-analyses report that the application of urease and nitrification inhibitors significantly reduced inorganic N leaching (−48%), N_2_O emission (−44%), and NO emission (−24%) (Burzaco et al., 2014; Qiao et al., 2015; Thapa et al., 2016) while increasing crop yield (7.5%) and NUE (12.9%) (Abalos et al., 2014). The beneficial effect of nitrification inhibitors may depend on the environment (e.g., soil pH and texture) and other management factors (e.g., irrigation and N fertilizer rate) (Abalos et al., 2014). The longevity of the inhibitors under soil conditions as affected by temperature is of key importance for their effectiveness (Menéndez et al., 2012; Guardia et al., 2018). Reaching the optimum balance between N oxides and greenhouse gas losses, N efficiency and crop yields often indicates the use of nitrification inhibitors with liquid organic sources such as manure slurries (Guardia et al., 2017). However, the use of nitrification inhibitors also increases cost, potential for NH_3_ emission and the risk of environmental contamination (Kim et al., 2012; Qiao et al., 2015). Recently, nitrapyrin has been detected in streams, suggesting off-site transport of this N stabilizing compound (Woodward et al., 2016) and DCD residues were detected in milk in New Zealand resulting in the suspension of DCD use in pastures (Thapa et al., 2016). Chemical nitrification inhibitors are not permitted in certified organic management systems, so organic alternatives are needed for management of nitrification and the use of neem seed oil (Opoku et al., 2014) has been suggested for this purpose.

#### Biological Nitrification Inhibition

Biological nitrification inhibition (BNI) is the ability of certain plant roots to impede soil nitrification through the production of biological inhibitors (Subbarao et al., 2013, 2017; Byrnes et al., 2017; Coskun et al., 2017). If BNI may be exploited to reduce nitrification in high nitrifying, low NUE systems then fertilizer use and loss may be decreased with associated decreases in GHG production. Some BNI's have been isolated from tropical pasture grasses that are adapted to low-N environments, in particular *Brachiaria* spp. have high BNI-activity in root systems and among field crops, sorghum (*Sorghum bicolor*) has been observed to produce biological nitrification inhibitors (Subbarao et al., 2015). Incorporation of these crops into rotations or pasture systems may help to retain N in these soils systems and increase soil N pools. If BNI traits from these plants could be transferred to grain crops, there may be potential benefits to NUE but unknown but likely tradeoffs to productivity. The search continues for biological nitrification inhibitors for the major grain crops especially maize.

Both plants and microbes may produce chemical compounds to inhibit nitrification to compete for ammonium in the rhizosphere. While most studies on BNI focus on plant root exudates; microbes could also produce compounds that inhibit nitrification. Soil microbes produce a wide array of signaling molecules and hydrocarbons including ethylene (Ladygina et al., 2006) that might be exploited for their inhibitory effects.

## Managing Nitrification Under a Changing Climate

The goal of reducing N losses from agricultural systems under changing climatic conditions is inherently complex spanning from technical through socio-economic approaches. Management that promotes shifting toward low nitrifying agricultural systems is part of a potential solution. Reducing the residence time and amount of inorganic N pools in agricultural soils while maintaining sufficient N fertility will require system based management. Reductions in the seasonal use of bare fallow, use of cover crops, increases in crop rotational diversity and perennial crops may increase the capacity for N retention in agricultural soils (Figures 5, 6). Unfortunately, projected impacts of changing climate may make our current mitigation efforts less effective (Le Roux et al., 2016; Bowles et al., 2018). Climate change affects nitrification in agriculture primarily through raising temperatures and the amount and intensity of rainfall (Robertson et al., 2013; Bowles et al., 2018). This combination of factors will increase the propensity for nitrification and subsequent N loss through leaching and denitrification. The controlling factors for nitrification described above have been used as drivers for the rate of nitrification in the process based models DayCent (Del Grosso et al., 2012) and DNDC (Li, 2007) (Table 1). In both of these systems nitrification is a function of ${\text{NH}}_{4}^{+}$ availability, water content, temperature, pH, and texture (Grant et al., 2016) although DNDC more explicitly drives microbial reactions by consideration of the redox balance in the soil and the volumetric fraction of the soil that is anaerobic (Li, 2007). Trait-Based models of nitrification predict that there may be changes in ammonia and nitrite oxidizer communities driven by global change contributing to feedback effects (Bouskill et al., 2012; Le Roux et al., 2016). Some factors that are under the control of land managers include: amount, form and application timing of N fertilizers, the use of nitrification inhibitors, and the amount and timing of water application in irrigated systems (Figure 5). These factors may be used to parameterize farm-scale (Del Grosso et al., 2016; Paustian et al., 2018) or trait-based models to advise management. However, factors such as the timing and intensity of rainfall, extreme drought events, and the timing of mineralization remain challenging management targets. In the future under a changing climate, elevated temperature and more variable precipitation will likely increase N mineralization and nitrification leading to even more urgent need to manage nitrification and prevent N losses from agriculture (Bowles et al., 2018).

## Summary and Future Directions

We review the controlling factors on the rate and extent of nitrification common in agricultural soils from temperate regions including substrate supply, environmental conditions, abundance and diversity of nitrifiers, and plant and microbial interaction with nitrifiers. Two main strategies for managing nitrification are to control ammonium substrate availability or inhibit nitrifiers directly. We propose four key future directions: (1) focus on enzymes involved in nitrification using proteomics—direct extraction of enzymes or fluorescently labeling key enzymes, (2) link ecophysiology in soil to sequence variants for trait-based modeling, (3) discover novel nitrification inhibitors, survey rootzone microbes and cultivars of major crop plants for inhibitory compounds, and (4) improve nitrification management modeling, especially for changing climate scenarios.

## Author Contributions

All authors listed have made a substantial, direct and intellectual contribution to the work, and approved it for publication.

### Conflict of Interest Statement

The authors declare that the research was conducted in the absence of any commercial or financial relationships that could be construed as a potential conflict of interest.
