# Supplementary material for: Controls and Adaptive Management of Nitrification in Agricultural Soils
Source: Front Microbiol. 2019 Aug 30;10:1931. doi: 10.3389/fmicb.2019.01931 (PMC6728921; doi:10.3389/fmicb.2019.01931)
Supplement: Supplementary file 1 [file Image_1.pdf]

Supplemental Figure 1. Relationship of a) soil temperature, b) soil water filled pore space and c) soil pH on functions used to control nitrification rates in DayCent from (Parton et al., 2001).

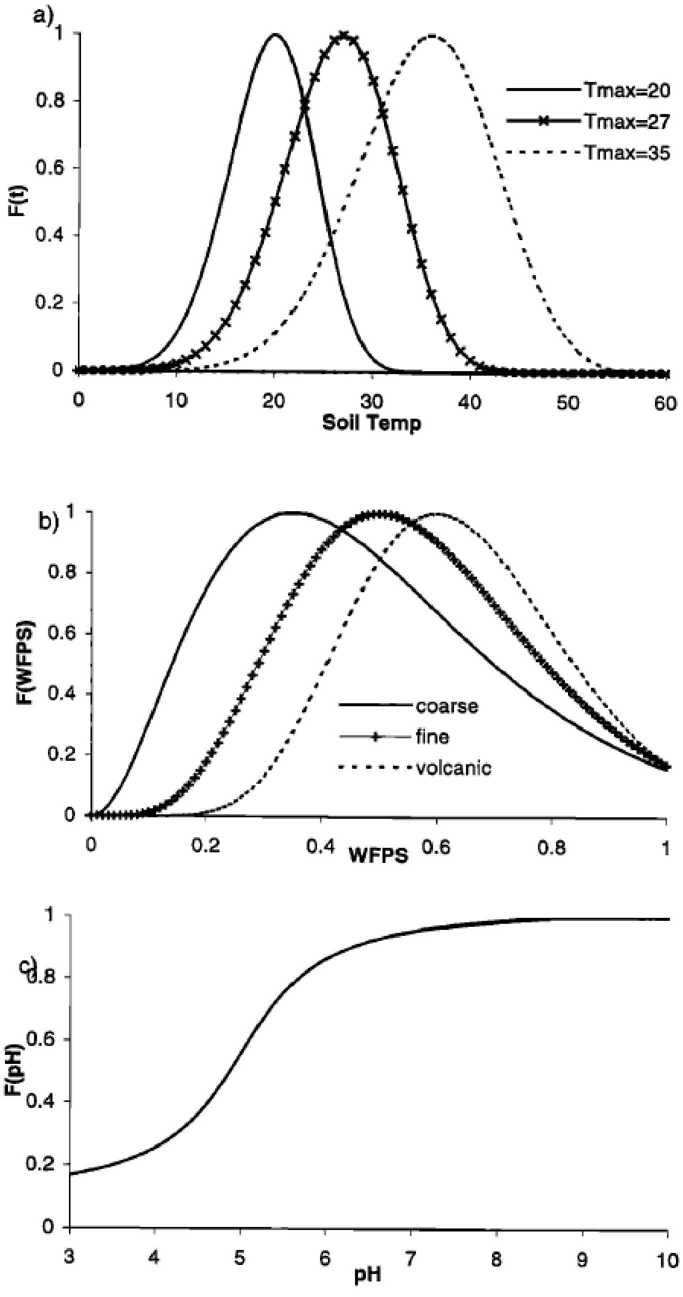

Parton, W.J., Holland, E.A., Del Grosso, S.J., Hartman, M.D., Martin, R.E., Mosier, A.R., et al. (2001). Generalized model for NOx and N2O emissions from soils. *Journal of Geophysical Research-Atmospheres* 106(D15)**,** 17403-17419. doi: 10.1029/2001jd900101.

AGU grants permission for individuals to use figures, tables, and short quotes from AGU journal and books for republication in academic works and to make single copies for personal use in research, study, or teaching provided full attribution is included. There is no need to request this permission from AGU.
